# Placental Mesenchymal Dysplasia Associated with Severe Intrauterine Growth Restriction: A Case Report

**DOI:** 10.30699/IJP.2023.548113.2828

**Published:** 2023-06-20

**Authors:** Arshia Yazdani, Mohammad Ranaee, Sara Babazadeh, Fatemeh Shafizadeh

**Affiliations:** 1 *Department of Pathology, Ayatollah Rouhani Hospital, Babol University of Medical Sciences, Babol, Iran*; 2 *Department of Obstetrics and Gynecology, Ayatollah Rouhani Hospital, Babol University of Medical Sciences, Babol, Iran*

**Keywords:** Fetal growth retardation, Placenta, Placental disease, Vascular disease

## Abstract

Placental mesenchymal dysplasia (PMD) is an uncommon placental lesion, which may mimic molar pregnancy at gross and microscopic examination. PMD can be associated with fetal growth restriction, Beckwith-Wiedemann syndrome, intrauterine fetal death, and preterm delivery. Nonetheless, it may also be associated with a normal appearing fetus.

We aimed to emphasize that clinicians, radiologists, and pathologists should be aware of PMD as one of the etiologies of intrauterine growth restriction (IUGR).

We presented the case of a 27-year-old gravida 1, para 1 woman who was admitted to Ayatollah Rouhani hospital, in Babol, Iran, at 30 weeks of gestation due to severe IUGR and fetal tachycardia. Ultrasound examination showed uteroplacental insufficiency and increased resistive index (RI) of umbilical artery. At last, a normal female fetus (1320 g) with no definitive anomalies was delivered by cesarean section. Pathological examination revealed cystically dilated stem villi with peripherally located thick-walled muscular stem vessels, and also stromal fibroblasts overgrowth in some stem villi. None of the examined sections revealed trophoblastic proliferation or stromal trophoblastic inclusion. The findings confirmed the diagnosis of PMD.

Careful radiological and pathological examination should be performed in the case of IUGR for ruling out the rare placental abnormalities, including PMD.

## Introduction

Placental mesenchymal dysplasia (PMD) is an uncommon placental anomaly characterized by enlarged placenta, aneurysmally dilated and congested chorionic plate vessels, and edematous stem villi without trophoplastic proliferation (1, 2). PMD was first described as stem villous hyperplasia in 1991 by Moscoso and colleagues (3). It can mimic molar pregnancy in ultrasound due to the presence of grape-like vesicles (4). Pathologically, PMD is a benign disorder of stem villi and is associated with elevated serum alpha-fetoprotein (5). The incidence of PMD is reported to be 0.02% per examined placenta. PMD can be associated with fetal growth restriction, Beckwith-Wiedemann syndrome, intrauterine fetal death, and preterm delivery. However, some cases of PMD show no fetus abnormality (6, 7), as three cases of PMD with live births of healthy newborns that were presented by Kong *et al*. in January 2020 (8).

In ultrasonographic and pathologic examinations, partial mole, complete molar pregnancy with twin pregnancy, and chorangioma with normal fetus have been recorded in differential diagnoses of PMD (9). Distinguishing the PMD from partial mole is very important, since management is different and misdiagnosis may lead to the termination of pregnancy for a healthy fetus with PMD (4).

According to a cohort study performed by Guenot *et al.* (2019), PMD was substantially underdiagnosed before delivery (10).

We hereby reported a case of PMD in a primigravid female who presented severe intrauterine growth restriction (IUGR) and fetal tachycardia and delivered a healthy baby girl.

## Case Report

A 27-year-old primigravid female was admitted to Ayatollah Rouhani hospital, Babol, Iran. She was at 30th week of gestation and referred to the hospital due to severe early IUGR and fetal tachycardia (fetal heart rate: 170 beat/minute). She had a history of polycystic ovary (PCO) and two years of infertility. She used Metformin and Letrozole prior to the pregnancy. She also used Aspirin from 18 weeks of gestation until 5 days before admission. Her previous ultrasonography for fetal anomaly and fetal echocardiogram test had yielded no abnormal finding. Ultrasonography shortly after admission showed uteroplacental insufficiency and increased resistive index (RI) of the umbilical artery. Significant laboratory findings at the time of admission were as follows: white blood count (WBC): 15200/µL, albumin: 2.7 mg/dL, RBC: 3.49×10^6^/µL, and hemoglobin (Hb): 9.6 g/dL. Doppler sonography was also performed that showed increased diastolic resistance, decreased umbilical artery flow and resistance, and an increased flow in the fetal middle cerebral artery, which indicated a brain-sparing effect. Biophysical sonography showed normal movement and fetal heart rate (8/8). Finally, cesarean section was performed and a live-born 31-week female infant was delivered. The infant birth weight was 1320 g. The Apgar scores at 1 and 5 minutes were 9 and 10, respectively. The delivered placenta was then sent for pathological examination. The premature infant was admitted to the Neonatal Intensive Care Unit (NICU). Echocardiogram, and cranial and urinary tract ultrasonography were normal. The infant was discharged after thirty days with rather good general conditions.

**Fig. 1:**
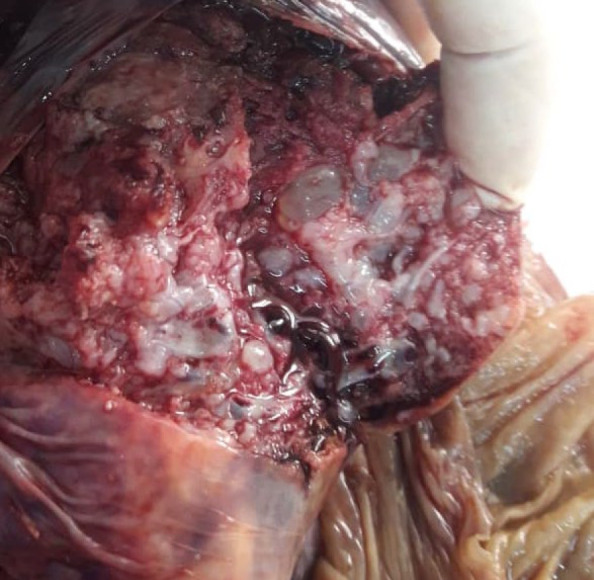
Gross image of enlarged placenta with multiple grape-like vesicles at maternal surface, especially around the cord insertion

In macroscopic evaluation, placenta was 12.5×5×4 cm and weighted 1000 g. The umbilical cord was eccentric and measured 17 cm in length and 1.4 cm in the greatest diameter, and included three vessels. Evaluation of serial sections of placenta noted numerous clusters of grape-like cystic vesicles containing a clear fluid ranging from 0.3 cm to 2.5 cm in diameter (Figure 1).

**Fig. 2.**
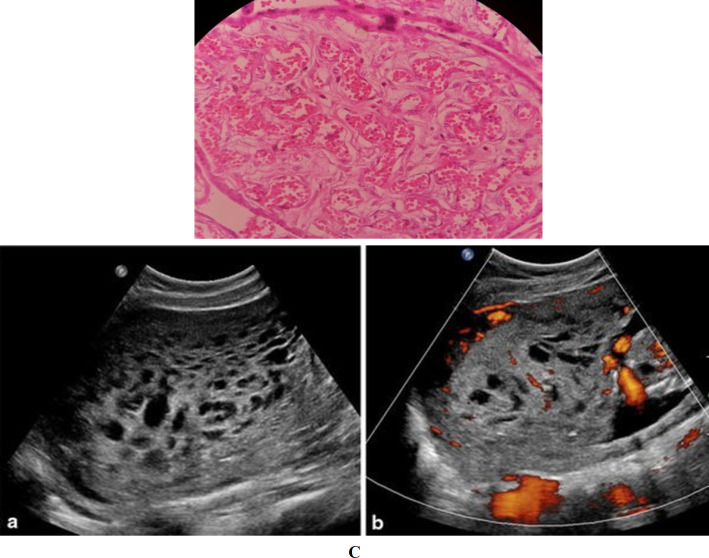
Photomicrograph of placenta. A: Large edematous stem villous and peripheral vessels. B: Stem villi with peripheral distribution of thick-walled vessels and increased stromal mesenchymal cells. C: Terminal villi with chorangiosis (H &E staining, 400x)

Microscopic examination, on the other hand, revealed enlarged, hydropic stem villi with peripherally located thick-walled blood vessels, and also stromal fibroblasts overgrowth in some stem villi. Terminal villi showed chorangiosis (Figure 2). Thrombosed vessels were seen in distal stem and intermediate villi. None of the examined sections revealed trophoblastic proliferation or stromal trophoblastic inclusion. These findings confirmed the diagnosis of PMD.

**Follow-up and Outcome**

On about 4-month follow-up, the mother and her infant found to be in good general conditions without any complaints or adverse events. 

## Discussion

PMD is an uncommon placental abnormality with a female:male ratio of 3.6:4.1. It is usually associated with IUGR (33%), preterm labor (33%), Beckwith-Weidmann syndrome (23%), and intrauterine fetal death (13%) (11, 12).

Infections, placental insufficiency, maternal hypertension, chorangioma, genetic abnormalities, uterus anomaly, advanced maternal age and PMD are described as etiologies of IUGR (10).

The causes of PMD are unknown. Different heterogeneous factors, including androgenic or biparental mosaicism, congenital mesodermal anomalies, hypoxia, and hypoperfusion with uncertain etiology may lead to fibroblastic overgrowth (9).

In this article, we reported a 27-year-old primigravid female with two risk factors for IUGR, including placental insufficiency and PMD. In May 2020, Sun *et al. *reported PMD in a healthy girl infant. In their case, cystic changes appeared in ultrasonography at 13 weeks of gestation and cystic changes progressively enlarged with progression of pregnancy (13). In our case, previous ultrasonography for fetal anomaly had represented no abnormality; while shortly after admission, uteroplacental insufficiency and increased resistive index (RI) of the umbilical artery were observed. Doppler sonography showed increased diastolic resistance, decreased umbilical artery flow and resistance, and increased flow in the fetal middle cerebral artery that were indicative of IUGR. In gross examination, we found numerous grape-like cystic vesicles containing a clear fluid, and PMD diagnosis that was made by histopathological examination of the placenta after delivery.

**Fig. 3.**
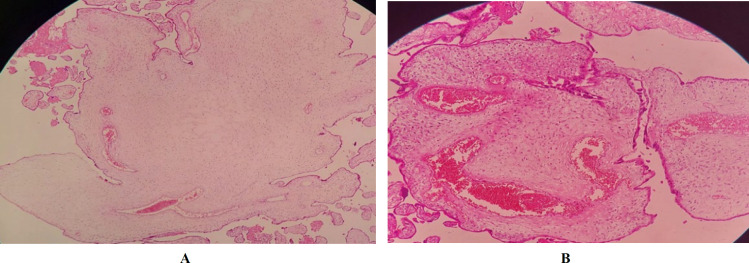
Ultrasonography and color Doppler ultrasound of the placenta with PMD. a: Multicystic hypoechoic areas “swiss-cheese” or “moth-eaten placenta” appearance . b: Different degrees of flow from the aneurysmally dilated vessels (14)

Various prenatal ultrasound findings can be traced in PMD. Placental thickening with multicystic hypoechoic areas: “Swiss-cheese” or “moth-eaten placenta” appearance are classic manifestations diagnosed by ultrasound examination. In color Doppler (stained-glass appearance), different degrees of flow from aneurysmally dilated vessels in some cases of PMD are seen (Figure 3) (14).

The diagnosis of PMD is often suspected in ultrasound evaluation but some laboratory findings such as serum human chorionic gonadotropin (HCG) and alpha-fetoprotein (AFP), karyotype analysis, and color Doppler sonography can assist in the diagnosis of PMD (15).

 The prognosis of pregnancy in PMD may be poor, however it may be associated with good pregnancy outcomes and lead to the birth of a healthy baby (16). In January 2021, Kodera *et al*. published the largest study on PMD cases with their clinical data. They collected the data of 49 cases of PMD from 2000 to 2018. They reported that the prevalence of fetal growth restriction (FGR), threatened premature delivery, fetal death, and hypertensive disorder of pregnancy in PMD were 34 (72.3%), 14 (29.8%), 8 (17.0%), and 6 (12.8%), respectively. Of 39 live births, 23 (59.0%) were associated with premature induction of labor or cesarean section due to obstetric indications related to FGR (17). Fortunately, in our case, the mother and her infant were in good general conditions without any complaints or adverse events.

In this context, we would like to emphasize that clinicians, radiologists, and pathologists should be aware of PMD as one of the etiologies of IUGR.

## Conclusion

Careful radiological and pathological examinations should be performed in the case of IUGR to rule out rare placental abnormalities, including PMD. Although some cases of PMD show no fetus abnormality, various congenital abnormalities, including Beckwith-Weidmann syndrome may be associated with PMD; therefore, the delivered infant must be appropriately examined and followed up.

## Conflict of Interest

There is no conflict of interests to be disclosed.
